# Role of PARP Inhibitors in Cancer Immunotherapy: Potential Friends to Immune Activating Molecules and Foes to Immune Checkpoints

**DOI:** 10.3390/cancers14225633

**Published:** 2022-11-16

**Authors:** Ornella Franzese, Grazia Graziani

**Affiliations:** Department of Systems Medicine, University of Rome Tor Vergata, Via Montpellier 1, 00133 Rome, Italy

**Keywords:** PARP inhibitor, BRCA, DNA damage response, immunotherapy, PD-1, PD-L1, CTLA-4, immune checkpoint inhibitor, combination therapy, cancer

## Abstract

**Simple Summary:**

This review aims at analyzing an emergent topic regarding the possible combined use of poly (ADP-ribose) polymerase (PARP) inhibitors (PARPi), anticancer drugs approved to treat tumors with defective repair of DNA damage, and immunostimulating agents, like Immune Checkpoint Inhibitors (ICIs), that represent the current treatment of a variety of malignancies. Preclinical investigation shows how PARPi, by positively impacting anti-tumor immune response through DNA damage-related mechanisms, might render cancer cells more responsive to immunotherapies, especially in the setting of genomic instability. In addition to well-recognized mechanisms that may be elicited by PARPi, recent experimental evidence is summarized further supporting the potential synergistic effects of PARPi and ICIs. We believe that an in-depth analysis of the tumor genome as well as of the characteristics of the tumor microenvironment at a single-patient level, along with an implementation of clinical trials that are presently at an early-stage, can contribute to identify more effective and individually-tailored treatments.

**Abstract:**

Poly (ADP-ribose) polymerase (PARP) inhibitors (PARPi) induce cytotoxic effects as single agents in tumors characterized by defective repair of DNA double-strand breaks deriving from BRCA1/2 mutations or other abnormalities in genes associated with homologous recombination. Preclinical studies have shown that PARPi-induced DNA damage may affect the tumor immune microenvironment and immune-mediated anti-tumor response through several mechanisms. In particular, increased DNA damage has been shown to induce the activation of type I interferon pathway and up-regulation of PD-L1 expression in cancer cells, which can both enhance sensitivity to Immune Checkpoint Inhibitors (ICIs). Despite the recent approval of ICIs for a number of advanced cancer types based on their ability to reinvigorate T-cell-mediated antitumor immune responses, a consistent percentage of treated patients fail to respond, strongly encouraging the identification of combination therapies to overcome resistance. In the present review, we analyzed both established and unexplored mechanisms that may be elicited by PARPi, supporting immune reactivation and their potential synergism with currently used ICIs. This analysis may indicate novel and possibly patient-specific immune features that might represent new pharmacological targets of PARPi, potentially leading to the identification of predictive biomarkers of response to their combination with ICIs.

## 1. Introduction

The PARP superfamily comprises 17 different molecules, characterized by distinctive activities [1,2]. PARP-1, the most representative member of the group, was originally recognized for its function of identifying and repairing DNA single-strand breaks (SSBs) [3]. PARP-1 exerts its activity through the post-translational modification of partner molecules by adding linear or branched poly(ADP-ribose) (PAR) chains using NAD+ as its substrate [4], thus modulating the functional engagement of enzymes involved in the repair of SSBs (i.e., base excision repair (BER)) and the activity of other repair systems [5,6,7,8,9,10]. Moreover, PARP-1 is involved in the regulation of other cellular processes, including gene expression, mitosis, apoptosis and sex hormone-mediated signaling [11,12]. Besides PARP-1, PARP-2 and PARP-3 are also involved in the repair of DNA damage, presenting a certain degree of redundancy in some of PARP-1 functions [13]. 

Inhibition of PARP activity has been shown to induce synthetic lethality in breast cancer gene 1 and 2 (*BRCA1/2*) mutated tumors, which are not able to repair DNA double strand breaks (DSBs) [8,14,15,16,17,18]. Remarkably, BRCA1 and BRCA2 preserve genomic stability and inhibit tumorigenesis by fostering DSBs repair via the homologous recombination (HR) system and are often mutated in hereditary breast and ovarian cancers [19]. Moreover, *BRCA1/2* mutations increase the risk for pancreatic and prostate tumors [20,21,22]. PARP inhibitors (PARPi) compete with NAD+ for the binding to the catalytic active PARP domain, inhibiting the synthesis of PAR chains with consequent impairment of chromatin structure remodeling and recruitment of DNA repair proteins at the damaged site. Thus, inhibition of PARP activity results in the generation of persistent SSBs that are converted into DSBs due to collapse of stalled replication forks during DNA replication [23]. In HR-defective cells, replication-associated DSBs cannot be effectively repaired and become lethal [18,24]. Furthermore, in the absence of an efficient HR-mediated repair of DSBs, aberrant activation of the error-prone nonhomologous end joining (NHEJ) takes place. NHEJ is an alternative DSBs repair system that directly re-ligates DNA broken ends without taking into account sequence homology. This leads to an inadequate repair of DSBs, contributing to genomic instability and tumor cell lethality [25]. In addition, PARPi can “trap” PARP-1/PARP-2 at DNA breaks due to inhibition of PARP auto-PARylation, thus preventing PARP protein release from the site of damage. Moreover, PARPi interaction with the NAD+ site allosterically increases the DNA binding of PARP N-terminal region. This leads to stalling of the replication fork and eventually cell killing mainly as a consequence of SSBs conversion into unrepaired DSBs [26,27,28]. The cytotoxicity based on this mechanism requires PARP-1 expression, since its silencing abolishes both PARPi trapping efficiency and cell killing effects, whereas PARP-2 (whose expression is much lower than PARP-1) has minimal influence on tumor sensitivity to PARPi [26].

The anti-tumor efficacy of PARPi as monotherapy, in the context of tumors expressing either germline or somatic mutations in the *BRCA* genes, has been established by pre-clinical and clinical investigations that have led to the FDA/EMA approval of four different drugs (i.e., olaparib, rucaparib, niraparib and talazoparib) for the treatment of advanced/metastatic ovarian cancer, triple negative breast cancer (TNBC), pancreatic cancer and prostate cancer [29,30,31,32,33]. Approved PARPi possess similar ability to inhibit the catalytic activity of PARP-1 and some of them also inhibit PARP-2 and PARP-3. Moreover, they markedly differ in PARP trapping activity with talazoparib being the most potent in this respect [34]. Consistently, talazoparib is the PARPi with the highest ability to induce cytotoxic effects in HR-deficient cells [35].

From the time PARPi have been approved [36,37,38], other genomic defects that result in altered HR function have been found to increase tumor susceptibility to these agents, leading to the extension of their use, in the case of ovarian cancer, regardless of the *BRCA* mutational status [39,40,41]. However, even in the presence of *BRCA* mutations or HR deficiency not all patients show a favorable response to PARPi with more than 40% of them experiencing treatment failure. For instance, the phase 3 SOLO-1 trial indicated that the 5-year progression-free survival (PFS) of patients with advanced, newly diagnosed, *BRCA* mutated ovarian cancer, treated with olaparib as maintenance monotherapy for up to 2 years, was about 50% (48% (95% CI 41–55) vs. 21% (14–28) in the placebo group) [42]. These data highlight not only the need of additional biomarkers to better select patients who might benefit from PARPi as monotherapy, but also the importance of identifying a solid biological rationale for their combination with agents endowed with a non-overlapping mechanism of action.

Immune checkpoint (IC) inhibitors (ICIs), working by antagonizing the immunosuppressive strategies engaged by tumor cells, have strongly impacted the outcome of cancer immunotherapy. Indeed, ICIs have renewed the therapeutic approach of several advanced/metastatic forms of cancer leading to the FDA/EMA authorization of anti-Cytotoxic T-Lymphocyte Antigen 4 (CTLA-4) (i.e., ipilimumab), Programmed Cell Death 1 (PD-1) (i.e., nivolumab, pembrolizumab, cemiplimab, dostarlimab), and Programmed Cell Death Ligand 1 (PD-L1) (i.e., atezolizumab, avelumab, durvalumab) monoclonal antibodies (mAbs). Approved indications include advanced/metastatic melanoma, non-small cell lung cancer (NSCLC) or small-cell lung cancer (SCLC), mesothelioma, esophageal squamous cell carcinoma, gastric cancer, hepatocellular carcinoma, urothelial carcinoma, cervical cancer, microsatellite instability-high (MSI-H) or mismatch repair deficient (dMMR) colorectal cancer, renal cell carcinoma, Hodgkin’s lymphoma, head and neck squamous cell carcinoma, Merkel-cell carcinoma, and tumor mutational burden-high cancers [43]. However, while showing significant efficacy, the clinical outcome of IC blockade may differ, with several patients not responding at any rate or finally showing resistance and tumor recurrence [44]. This strongly advocates the requirement of effective combined approaches to overcome resistance.

Remarkably, the efficacy of IC blockade has been shown to be largely conditioned by neoantigen expression and recognition [45,46,47,48], suggesting that the permanence of DNA alterations deriving from PARP inhibition could support ICI-mediated boosting of the antitumor response. Interestingly, tumors sensitive to PARPi as a result of deleterious germline or somatic mutations in the *BRCA* genes have been frequently described as poorly responding to IC blockade single treatment [49,50]. Moreover, PARPi have demonstrated to control the tumor immune microenvironment (TME) by modulating both cancer cell genomic instability and T cell mediated responses. These observations have provided the rationale for testing the combined inhibition of PARP and ICs for the management of selected tumors.

In this review, we aim to survey recognized as well as potential mechanisms elicited by the inhibition of PARP-1 that could support the immune re-invigoration promoted by currently employed ICIs. In particular, we have summarized the established mechanisms underlying the synergistic effect deriving from PARP inhibition and IC blockade. Furthermore, we have investigated additional unexplored mechanisms potentially elicited by PARP-1 inhibition that could provide support to the T cell stimulation induced by ICIs, along with their intrinsic limitations. Finally, we have discussed early data from ongoing clinical trials of the combined approaches, from which we can also obtain essential information on potential biomarkers predictive of response that might be useful for patient selection.

## 2. Influence of PARP Inhibition on Immune Response to Tumors

### 2.1. Well Established Effects of PARP Inhibition on the Different Features of TME

The influence of PARPi on anti-tumor T cell immune response and on TME features can be mediated by different mechanisms. Some of them are directly related to the defective DNA damage repair, others are instead associated with the activation of still not fully clarified pathways, which ultimately convert an immunologically silenced “cold” TME into an immunologically “hot” milieu, characterized by effective CD8+ T cells, type I macrophages (M1) and natural killer (NK) cells (see below).

As indicated above, inhibition of PARP activity induces cell death preferentially in *BRCA1/2* mutated or otherwise HR-defective tumors which are unable to repair DNA DSBs [30,51]. In fact, besides *BRCA1/2* mutations, a variety of additional genetic alterations may contribute to induce in cancer cells a “BRCAness phenotype”, characterized either by a defective DNA Damage Response (DDR) [52] or by reduced expression of other factors involved in DNA repair (e.g., PI3-kinase-related protein kinases Ataxia-Telangiectasia Mutated (ATM), RAD3-related (ATR), Checkpoint Kinase 1 and 2 (CHK1/2), DSS1, RAD51, CDK12, TP53, or Phosphatase and Tensin Homolog (PTEN)) [52,53,54,55]. Therefore, these molecular features may represent responsive predictive biomarkers for PARPi-based treatment of non-*BRCA* mutated tumors [55,56,57].

On the other hand, among the first evidence about cancer responsiveness to ICIs, a phase 2 investigation showed that the efficacy of the anti-PD-1 pembrolizumab was superior in solid tumors with defective MMR enzymatic activity, and that the clinical outcome was related to the extent of somatic mutations, with best patient’s response occurring in the presence of high frequency of neoantigen-specific T cells [58].

DDR defects, including those affecting *BRCA1/2*, are related to greater tumor mutational burden in tumors, including ovarian cancer and NSCLC [59,60], contributing to increase the amount of tumor-specific neoantigens, both valuable biomarkers for predicting the efficacy of ICI therapy, in combination with other indicators [61,62,63,64,65,66,67,68].

Therefore, the relationship existing amid tumor DNA damage and an improved immune response has suggested a potential role for PARPi as sensitizers of tumor cells to ICIs [69,70], by impairing DNA repair and producing genomic instability with consequent expression of neoantigens that could support the re-invigoration of anti-tumor T cell mediated response.

Neoantigen presentation provided by antigen-presenting cells (APCs), including dendritic cells (DCs), monocytes/macrophages and B lymphocytes, in the context of major histocompatibility complex class I (MHC I) molecules, is required to stimulate the cytotoxic response of CD8+ T cells. Of note, MHC I expression and antigen presentation are improved by DDR [60]. PARP inhibition itself has been shown to stimulate both MHC I expression, following activation of ATM/ATR kinases [71,72], and DDR, alongside immunogenic cell death (ICD), thus stimulating the damage-associated molecular patterns (DAMPs) to further support the engagement of APCs [73].

Inhibition of PARP-1 increases the extent of DNA damage and the release/accumulation into the cytoplasm of DNA fragments. These can be sensed by innate anti-viral DNA mechanisms, including the cyclic GMP–AMP synthase (cGAS)/stimulator of interferon (IFN) genes (STING) which releases the STING carboxyl terminus to subsequently recruit and activate by phosphorylation the TANK-binding kinase 1 (TBK1) and the IFN regulatory factor 3 (IRF3) [74,75,76]. The cGAS/STING/TBK1/IRF3 pathway is responsible for the activation of type I IFN response [69,77,78,79]. In addition, activation of IRF3 is required to produce inflammatory cytokines including IFN-γ, TNF-α, IL-6 [80,81,82], and IL-12, of which the latter is critical in promoting the T cell-DC crosstalk [83] required to promote antigen presentation. Other released proteins include C-X-C motif chemokine ligand (CXCL)1, CXCL2, CXCL9, and CXCL10 that support both development and tumor infiltration of macrophages and CD8+ T cells [80]. STING also stimulates the activation of NF-κB, which collaborates with IRF3 in promoting the release of pro-inflammatory cytokines such as IL-1β, IL-6 and IFN-γ [84,85,86] Furthermore, although type I IFNs are mainly produced by DCs in the TME, a paracrine cGAS/STING pathway stimulation has been observed in neighboring DCs, exclusively in BRCA1-deficient models of TNBC and ovarian cancer, mediated by pro-inflammatory cytokines release and possibly tumor DNA exocytosis [87,88], according to the described role played by extracellular DNA in the generation of an inflammatory phenotype [89]. Thus, based on the different trapping efficiency and ability to induce DNA damage and generate DNA fragments of the various clinically relevant PARPi, it can be hypothesized that PARPi with strong trapping activity (e.g., talazoparib) will more efficiently synergize with ICIs than weak PARP trappers (e.g., veliparib).

Of note, improved antigen presentation and increased frequency of tumor associated DCs with high expression of CD40, CD80, and CD86 co-stimulatory molecules have been described in response to olaparib, together with enhanced transition of CD8+ T cells to the tumor site [90]. The CD40/CD40L engagement has been shown to positively influence both humoral and cellular immune responses [91]. Moreover, the increased CD80/CD86 expression induced by olaparib holds additional implications. In fact, besides representing a critical player in T cell co-stimulation, CD80 forms heterodimers in cis with PD-L1, reducing its own interaction with CTLA-4, while maintaining the ability to activate CD28 [92].

Despite possessing a very high mutational burden, SCLC is frequently characterized by severe immunosuppression and poor intra-tumor T-cell infiltration [93]. According to these observations, clinical studies exploring the use of anti PD-1 or PD-L1 mAbs in SCLC patients have revealed poor clinical outcome [94]. SCLC over-expresses PARP-1 [95], whose inhibition was found to up-regulate intra-tumor T cell infiltration and to synergize with PD-L1 blockade, provoking substantial tumor decrease in mouse models [96]. Conversely, olaparib and anti–PD-L1 single treatments did not induce marked antitumor effects. Remarkably, knockdown experiments have unequivocally established the direct role of the STING/TBK1/IRF3 pathway in the antitumor immune response stimulated by the combined treatment in SCLC [96].

Increasing evidence suggests that the distribution, density, and phenotype of tumor infiltrating lymphocytes (TILs) positively affect the efficacy of ICIs [97]. Different studies have demonstrated that PARP inhibition up-regulates intra-tumor infiltration by both CD4+ and CD8+ T cell subsets [87,98]. In particular, Strickland et al. established that BRCA1/2-defective high-grade serous ovarian cancers exhibit significantly higher whole CD3+ as well as CD8+ tumor T cell infiltration and better patients’ survival outcome compared with HR-proficient tumors [59]. Of note, olaparib-induced increase in terms of CD8+ T cell recruiting in the TME has been shown to be mediated by the tumor cGAS/STING pathway, an effect more obvious in HR-defective than in HR-proficient TNBC cells [87]. The preferential stimulation of STING/TBK1/IRF3 pathway in BRCA1-defective tumors has been confirmed by further in vivo studies [99], speaking in favor of a prediction for greater intra-tumor T cell penetration in BRCA1-defective cancers and of a more modest effect of combined therapy in tumors with a proficient HR system [86]. 

Conversely, according to other studies [100], PARP inhibition has been shown to induce cytosolic DNA accumulation and type I IFN response, along with increased intra-tumor CD8+T cell infiltration dependent on C-C chemokine ligand 5 (CCL5) and CXCL10 chemokines, irrespective of the *BRCA* mutational state. These observations foster the crucial issues of whether: (i) a defective BRCA is strictly required for the STING-dependent immunological improvement associated with PARPi, and (ii) additional mechanisms are involved beside those induced by unsolved genomic defects. However, in BRCA competent cancers, characterized by a possibly reduced STING-associated IFN type I pathway, where DNA lesions induced by PARP blockade could be inadequate, the use of a STING agonist combined with IC blockade has been suggested as an alternative potential therapeutic combination approach [87,101].

It is also well acknowledged that modulation of the intra-tumor T cell equilibrium toward a CD4+Th1 response is critical to promote cytotoxic CD8+ T cell functional activity and an effective anti-tumor response [102]. CD4+ Th1 effector cells, by producing key cytokines such as IFN-γ, TNF-α, and IL-2, are critical players in anti-tumor protection, able to mediate cancer cell elimination through activation of both innate and adaptive immunity [103]. In the TME, IFN-γ improves the immunogenicity of tumor cells by increasing the expression of MHC class I and II, which makes them more susceptible to recognition by effector cells [104]. Moreover, IFN-γ enhances the tumoricidal action of M1 macrophages and the recruitment of NK and T cells from the periphery to the tumor site via CXCL9 and CXCL10 chemokines. Remarkably, impairment of PARP-1 activity has been shown to bias T cell phenotype balance by favoring Th1 subset differentiation while lowering the Th2 specific immune response [105]. This effect has been possibly related to calpain-mediated degradation of the transcription factor STAT6, which is required for Th2 differentiation signaling [106]. An additional mechanism proposed for PARPi-associated inhibition of Th2 responses is provided by the reduced expression of the transcription factor GATA-3 [107], the main regulator of IL-4/IL-5/IL-13 cytokine production in activated human CD4+ T cells [108]. PARP-1 has also a recognized role in the epigenetic control of gene expression by inducing chromatin remodeling through histone poly(ADP-ribosyl)ation (PARylation) and modification of the DNA methylation status [109]. Since epigenetic changes may promote Th2 cell differentiation by modulating IL-4 gene transcription [110], it cannot be ruled out that PARPi might favor a Th1 response also through this mechanism. Therefore, PARP inhibition-mediated effect on the fine-tuned balance underlying CD4+ Th differentiation within the TME represents an additional mechanism potentially improving the outcome of cancer immunotherapy.

Nevertheless, PARP-1 inhibition has shown to modulate the differentiation of CD4+ T cells also by favoring the development of immunosuppressive regulatory T cells (Tregs). This effect is mediated by a decrease in the transcription factor FOXP3 destabilization, which is normally induced via PARylation [111,112], with consequent increased expression of genes controlled by FOXP3, such as those coding for CD25, CTLA-4 and interleukin 10 (IL-10). In mouse Tregs deficient for PARP-1, FOXP3 has been found at conserved non-coding DNA regions, a critical mechanism required for preserving FOXP3 gene expression in this cell subset [113]. Accordingly, treatment with the PARPi talazoparib has been correlated to increased Treg infiltration within the tumor site [98].

Conversely, notwithstanding a considerable up-regulation in terms of whole intra-tumor CD4+ T cell frequency, a remarkable change in immunosuppressive CD4+FOXP3+ Tregs was not observed in response to olaparib by Pantelidou et al. [87]. The authors suggested that further studies are required to establish the consequences of PARP blockade both in terms of overall impact on CD4+ T cell infiltration at the tumor site and of the role of different CD4+ T cell subgroups in the antitumor efficacy of PARPi. Moreover, in a preclinical in vivo model of SCLC, the tumor impairment observed with the combined use of PARPi and anti-PD-L1 was accompanied by reduced CD4+FOXP3 Treg frequency and amplified cytotoxic CD8+ T cell response [96].

Overall, the contrasting results reported suggest the requirement of further investigation to define the outcome of PARP inhibition on Treg development and function and the potential negative impact on ICI-mediated reactivation of T cell functionality in different cancer settings. Moreover, Treg induction mediated by PARPi can prevent the inflammatory response and the systemic toxicity potentially deriving from sustained STING pathway activation as well as by ICI co-administration.

Remarkably, a direct crosstalk has been also identified between PARP-1 and PD-L1/PD-1 signaling pathway. Although not completely adequate for all tumor types, PD-L1 expression is still considered as one of the main biomarkers for predicting clinical response to anti-PD-1 mAbs [114]. Cumulative indications have suggested that PARPi can increase PD-L1 expression, likely through the activation of DNA damage-related STING and the downstream TBK1-IRF3-type I IFN signaling [115] or via the ATM-ATR-CHK1 pathway [116,117]. Moreover, inhibition of PARP-1, either by gene knockdown or pharmacological treatment with olaparib or talazoparib, has been shown to increase PD-L1 expression by deactivating the GSK-3β kinase, which promotes its proteasomal break-down [118,119], irrespective of the *BRCA* mutational status. Indeed, an increase in GSK3α/β phosphorylation at Ser21 and Ser9, which characterizes the inactive form of the kinase, has been observed [120]. As a result, despite being directly responsible for restraining T cell response, the increase in PD-L1 expression mediated by PARPi can amplify tumor sensitivity to anti PD-L1 mAbs.

On the other hand, additional interference of PARPi with ICs-mediated signaling has been also demonstrated in preclinical mouse models of BRCA1-deficient ovarian tumors showing reduced expression of PD-1, Lymphocyte-activation gene 3 (LAG-3), and T-cell immunoglobulin (TIM-3), after combined PARPi and PD-1 blockade, resulting in reactivation of T cell functionality [98,121,122,123]. 

A potential drawback related to the increase in pro-inflammatory cytokines deriving from PARPi-mediated DNA damage could be represented by an enhancement of cancer initiation, promotion, and metastatic capacity [124]. In this context, a critical role may be played by the PARPi-mediated release of the inflammatory chemokine CCL5 in the TME [87]. In fact, in addition to favoring tumor infiltration of CD8+ T lymphocytes [87], the activation of the CCL5/CC type 5 chemokine receptor (CCR5) axis is involved in the development and progression of several malignancies, including breast and ovarian cancer, and in the generation of an immunosuppressive TME [125,126,127,128]. In particular, CCL5 has been shown to support the αvβ3 integrin-mediated tumor cell migration through activation of the PI3K/AKT and NF-kB signaling pathways [129], and by increasing the production of matrix metallopeptidases 2 (MMP-2) and MMP-9 [130,131], able to promote tumor cell invasiveness. Moreover, although being able to impair osteosarcoma growth, PARPi have been shown to support its metastatic potential through phosphorylation of the ezrin protein [132], a cytosolic structural molecule that regulates cell growth and motility [133]. This observation is of relevance because a high expression of the ezrin protein has been related to lower overall survival (OS) [134] and disease progression in patients with ovarian cancer [135], unveiling a potential mechanism of resistance to PARPi.

### 2.2. PARP-1 Inhibition Favors NKG2D Activity

To provide the rationale for innovative combined therapeutic strategies effective in the context of diverse tumor immune landscapes, the identification of additional mechanisms underlying the immunostimulating effects of PARPi is strongly required.

Type II transmembrane receptor Natural Killer Group 2 Member D (NKG2D) is a stimulatory receptor expressed by many immune cell subsets, including NK and CD8+ T cells, but also γδ and CD4+ T lymphocytes in definite settings, which promotes cytotoxic responses against target cells expressing its specific ligands. Although both human CD8+ T and NK cells express NKG2D [136,137,138,139], its role within these two distinct sub-populations is different. While in NK cells, the signaling pathway elicited by NKG2D is sufficient to induce killing of target cells [138], in CD8+ T cells, NKG2D delivers a co-stimulatory signal competent to support T cell receptor (TCR) activation [137,138,140,141,142,143], (although through distinctive pathways from those activated by the canonical CD28 co-stimulatory molecule) [144]. Of note, unlike CD28 which is represented within 50% of CD8+ T cells [145], NKG2D is ubiquitously expressed among this population, potentially representing an effective co-stimulatory alternative target in CD8+ T cells that lose CD28 following terminal differentiation driven by continuous stimulation [146]. Moreover, since the specific NKG2D ligands are more expressed than the CD28 counterparts CD80 and CD86, only present on APCs, NKG2D represents a valid co-receptor to be exploited in immunological approaches for cancer treatment.

Distinctive NKG2D ligands include MHC-I-related chain (MIC) A/B and members of the ULBP family (UL16 binding proteins 1–6 in humans) [147]. In normal settings, NKG2D ligands are moderately expressed [148,149], but are promptly stimulated by stress conditions, including viral infection, cancer, DNA-damage, TLR signaling and cytokine-induced cell proliferation [140,147,148]. Although increased in stressed cells, NKG2D ligands are detected in normal tissues, including spleen, skeletal muscle, and skin [150]. Moreover, besides its co-stimulatory role, NKG2D is involved in the process of T cell memory progression. Of note, memory CD8+ T cells can be activated by cytokines under definite settings, which induce a specific NK-like killer phenotype, characterized by the acquisition of cytotoxicity towards tumor cells via NKG2D activation without requirement of TCR engagement [143,151].

The role of NKG2D in immunosurveillance against cancer has been demonstrated by data obtained in animal models showing that its deficiency leads to a reduced ability to fight tumors while the presence of its ligands has been shown to confer an effective barrier to cancer progression [140,148]. Although both primary tumors and metastases often accumulate MICA, MICB, and ULBP proteins [152], low or absent expression of NKG2D ligands represents a well-established strategy employed by leukemia cells for escaping the immune response [153,154]. Therefore, induction of NKG2D ligand expression may represent an appealing goal to be achieved to foster the impact of immunotherapies in cancer treatment.

PARP-1 has been implicated in the negative control of the NKG2D ligands, MICA and MICB. Frequent relapse in acute myeloid leukemia (AML) has been ascribed to the persistence of leukemia stem cells (LSCs). These cells strongly contribute to drug resistance [155,156] and are characterized by poor expression of NKG2D ligands [157], which makes them distinctively less vulnerable as compared with bulk AML cells to the NKG2D-dependent effector functions. Of note, inhibition of PARP-1 has been shown to promote NKG2D ligands expression on LSCs, without affecting their expression on non-stem tumor or normal hematopoietic cells in a preclinical AML mouse model [158], providing a rationale to combine PARPi and NK cell-based immunotherapy for AML treatment [143].

CAR-T (chimeric antigen receptor T) cell therapies use genetically engineered T cells to contrast malignancies and have been proven beneficial in in patients with refractory B cell malignancies [159]. Most of the NKG2D-based CAR constructs are realized in T lymphocytes characterized by TCR heterodimers α and β, and comprise an extracellular, full-length human NKG2D linked to an intracellular CD3ς signaling domain [160]. Notably, the use of NKG2D-CAR T cells is currently being tested in the clinical practice, and data on NKG2D-based CAR clinical trials along with possible drawbacks have been recently provided [161].

However, expression of NKG2D ligands on normal cells, as a consequence of the activation of DNA repair mechanisms, may pose safety issues, due to the lethal toxicity possibly produced by off-tumor effects. In fact, preclinical mouse investigation has shown how adoptive transfer of NKG2D-CAR T cells can potentially promote fatal toxicities, although the severity of toxic effects was different depending on the mouse strain tested [162]. Overall, these observations suggest a possible use of PARPi in combination with NKG2D-based therapies, following careful consideration of the highly heterogeneous expression of NKG2D ligands either by cancer or normal cells.

**Figure 1:**
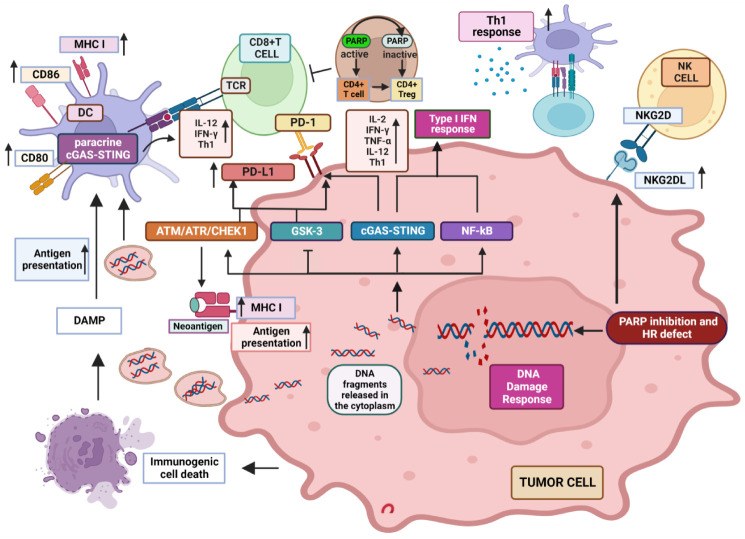
Recognized modifications induced by PARP inhibition in the TME. PARPi impair DNA repair which produces DNA fragments that are released in the cytoplasm, where they activate cGAS inducing STING pathway and the generation of a type I IFN inflammatory response. NF-kB is also activated, contributing to the production of inflammatory cytokines. DNA fragments can also be released extracellularly inducing paracrine GAS/STING response in DCs and increasing the expression of MHC molecules as well as antigen presentation. DDR response induces MHC expression in tumor cells, improving tumor-associated neo-antigen presentation induced by PARPi. PD-L1 expression is up-regulated via cGAS/STING, ATM/ATR pathway and GSK3 inactivation, potentially increasing the response to ICIs. PARPi induce NKG2D ligand expression, enhancing NK and CD8+ T cell anti-tumor cytotoxicity. See text for a more detailed explanation. “Created with BioRender.com.”

Key recognized modifications induced by PARPi in the TME are represented in Figure 1.

## 3. What the Future Holds: Identification of Additional Targets Modulated by PARPi Supporting Their Combination with ICIs

### 3.1. PARPi Positively Impact CD155 Expression: A Delicate Balance between T Cell Co-Stimulation and Co-Inhibition

Of note, a novel PARPi, AMXI-5001, has been shown to stimulate the expression of poliovirus receptor (PVR, CD155, necl-5) [163], an adhesion molecule belonging to the nectin/nectin-like family, often overexpressed on tumor cells and involved in many different processes such as cell adhesion, migration, and proliferation [164]. In contrast to these pro-tumorigenic functions, CD155 may stimulate NK and T cell-mediated responses through the binding to CD226 [165], a functional protein member of the immunoglobulin superfamily, expressed on NK and T cells, monocyte/macrophages (mainly in inflammatory monocytes where it plays a critical role in adhesion to CD155-expressing cells) [166] and DCs, among others [167]. CD226 has been involved in anti-tumor NK cell and T cell-mediated cytotoxicity [168] by contributing to the establishment of a strong interaction with tumor cells through the engagement of CD155 [169,170] that is required for an effective killing of tumor cells and inhibition of their metastatic spreading [171].

In naïve T cells, while barely represented in the absence of stimulation, CD226 is induced following activation with anti-CD3 plus anti-CD28 mAbs [172] or TCR stimulation [173]. Of note, CD226 has been shown not only to promote Th1 responses but also to impair Th2 function, and accordingly its inhibition has been associated with higher STAT-6 phosphorylation and GATA3 expression, along with a substantial increase in Th2 cytokines [174]. Based on these findings, it is possible to hypothesize that PARPi further potentiate Th1 responses by increasing the engagement of CD226 through an increased expression of its ligand CD155 on tumor cells. 

Among the distinctive developmental stages of murine NK cell progenitors, CD226 is already expressed in stage B immature NKs [175]. In humans, CD226 colocalization and coordinated regulation with lymphocyte function-associated antigen 1 (LFA-1) at the lytic immune synapse, is associated with the NK differentiation state and has been shown to be critical in providing support for the development of “educated” NK cells and to improve their function [176,177]. These observations have suggested that CD226 may represent a marker for mature NK cells [178,179]. After the binding of the specific ligands, CD226 interacts with the locally accumulated LFA-1 in the context of the immunological synapse [180] through phosphorylation at the S326 residue mediated by Protein kinase C (PKC). This interaction culminates with the binding of LFA-1 to the intercellular adhesion molecule 1 (ICAM-1) and the recruitment of Fyn kinase which phosphorylates the Y319 residue of CD226 [180], a modification capable of activating the extracellular signal-regulated kinase (ERK) and AKT. The latter, in particular, supports the cytotoxic activity of both NK and T cells [181,182,183]. Of note, CD226 expression can be modulated also by selected anti-tumor treatments. In particular, CD226 has been shown to be induced by bortezomib on both NK cells and γδ T cells [184] resulting in an improved cytotoxic activity against multiple myeloma cells. These findings clearly indicate that CD226 is a critical player strongly influencing the antitumor immune responses. Indeed, tumor-infiltration by CD8+ T cells with poor CD226 expression and high levels of the other specific ligand of CD155 (T cell immunoreceptor with Ig and ITIM domains, TIGIT), is associated with T cell exhaustion and tumor progression [185,186,187]. TIGIT co-inhibitory molecule is highly expressed on NK cells, CD8+ T cells and Tregs within the TME [188,189,190,191], whereas CD155 is widely expressed on tumor cells [192,193] and activates TIGIT signaling to dampen NK/T cell activation and development of an effective anti-tumor response [194,195]. Interestingly, TIGIT’s immunomodulatory effects have been found to be dependent on CD226 [195]. In particular, the anti-tumor activity of a combination of anti-PD-L1 and anti-TIGIT mAbs has been shown to be abolished by CD226 blockade [195]. Moreover, inhibition of TIGIT positively impacts mainly self-renewing CD8+ T cells characterized by high expression of CD226. Anti-TIGIT treatment selectively affects CD226hiCD8+ T cells by inducing its phosphorylation at tyrosine 322 [196], an observation which has encouraged the authors to propose high CD226 expression as a predictive biomarker of response to anti-TIGIT treatment. The competition between TIGIT and CD226 for the binding to CD155 partially contributes to explain their interdependence [197], because TIGIT also directly affects the intracellular CD226 signalling, by preventing its homodimerization, essential for the interaction with CD155 [195]. Accordingly, either the absence or poor CD226 expression on T cells can favor the engagement of CD155 with TIGIT, thus impairing T cell mediated anti-tumor response through different mechanisms: (i) inhibition of the AKT-mTORC1 pathway, with stabilization of FOXP3 and preservation of Treg immunosuppressive function [198] and (ii) recruitment of Src Homology 2-containing Inositol Phosphatase 1 (SHIP1) [199]. Therefore, an accurate assessment of the intra-tumor T cell expression of TIGIT along with that of CD226, which directly competes for the binding to CD155, is critically required at the single patient level to fully take advantage from PARPi-mediated increase in CD155 expression on tumor cells.

Of note, the PARPi AMXI-5001 has been shown to stimulate also the expression of death receptors DR4/5 [163]. DR4/5 selectively induces apoptosis in tumor cells throughout the engagement of TNF-related apoptosis-inducing ligand (TRAIL), expressed by NK cells. Accordingly, pre-clinical in vitro studies have shown that AMXI-5001 exerts synergistic activity with TRAIL in several tumor cell models, advocating that the antineoplastic outcomes of PARPi might involve an improved tumor sensitization to NK cell mediated-apoptosis by TRAIL stimulation.

### 3.2. PARPi Hamper the Consumption of NAD+: Potential Role in the Anti-Tumor T Cell Function

Besides the nature of stimulus, both the extent and persistence of T cell response are regulated by several cellular mechanisms, which prevent unwanted tissue damage related to uncontrolled inflammatory response. Alongside the numerous co-inhibitory receptors, such as PD-1, CTLA-4, LAG-3, TIM-3, and TIGIT [191,200,201], other mechanisms may negatively affect T cell activation, either by the induction of downstream inhibitory signals or by restraining the required metabolic modifications, limiting proliferation, and suppressing the release of inflammatory molecules [202]. Ectonucleotidases, able to control the extracellular amount of nucleotides, are critical regulators of T cell activation by producing adenosine that fosters an immunosuppressive TME [203,204]. Differently from CD39 and CD73, which generate adenosine through consecutive conversion from ATP to AMP and then adenosine [205], CD38 belongs to an alternative adenosine producing ecto-enzymatic pathway, involving CD203a/ENPP1 and CD73. CD38 has recently gained reputation as essential modulator of T cell activation and function potentially acting as an IC [206,207,208]. Accordingly, a phase 1 dose escalation and expansion study with an anti-CD38 Ab, CID-103, is currently recruiting previously treated, relapsed or refractory multiple myeloma patients (ClinicalTrials.gov Identifier: NCT04758767).

CD38 belongs to the nicotinamide adenine dinucleotide (NAD+) glycohydrolase/adenosine 5′-diphosphate-ribosyl cyclase family. Its activity results in the cyclization of NAD+ to cyclic ADP-ribose (cADPR) and hydrolysis of the latter to form ADP-ribose [209]. Thus, CD38 can reduce the extent of available NAD+ and affect the function of several NAD+-dependent molecules including PARP-1 and SIRT-1, a NAD-dependent deacetylase with multiple regulatory roles [209,210,211,212], thus playing a critical function in the control of T cell destiny and metabolic fitness [211]. A similar effect can be postulated for PARP-1: indeed, acute activation of PARP-1 by DNA damage or progesterone analog R5020 hormone stimulation might induce a strong reduction in total intra-cellular NAD+ [213,214]. This NAD+ deprivation has been observed especially in the case of sustained PARP-1 stimulation following prolonged stress able to alter cellular metabolic equilibrium [210], leading to severe ATP depletion [214]. This would result in a shift toward oxidative phosphorylation over glycolysis [215], which can negatively affect CD8+ T cell antitumor function that highly relies on a glycolytic metabolism [216].

Several studies have clarified some of the mechanisms involved in T cell inactivation consequent to NAD+ depletion, based on metabolic reprogramming [217]. Remarkably, TILs, often characterized by an exhausted phenotype, show significantly lower NAD+ as compared to peripheral blood derived lymphocytes (PBL). This finding suggests that T cell depletion observed with the transition from the periphery to the TME can be also ascribed to NAD+ deprivation, which reduces the number of mitochondria, ATP levels and respiratory capacity of T cells [218,219]. Accordingly, preclinical models testing CAR-T and PD-1 blockade have confirmed that exposure to NAD+ improves anti-tumor T cell cytotoxicity [219]. Therefore, strategies aimed at restoring NAD+ normal levels have been proposed as adjuvant therapeutic approaches to improve the clinical outcome of adoptive T cell therapy in patients with solid tumors. Based on these assumptions, PARPi can potentially promote T cell activation also by reverting the PARP-1-dependent consumption of NAD+, either alone or in cooperation with anti-CD38 mAbs [211].

At variance with these findings, studies on cADPR generated by CD38 for intracellular Ca2+ mobilization [220,221], indicated a possible positive role for CD38 in T cell activation [222], given the unequivocal role of Ca2+ signaling in triggering T cell stimulation. Indeed, CD38 expression has been shown to describe a subgroup of CD8+ T cells reinvigorated by anti-PD-1 blockade in human lung cancer [223]. In this scenario of CD38-associated T cell stimulation rather than impairment, PARPi could indirectly contribute to preserve the intra-cellular NAD+ levels required for overall T cell functionality and CD38-associated cADPR generation [211], which may enhance the response to anti PD-1 mAbs [224].

Considering the role of NAD+ in improving the anti-tumor activity of canonical and engineered T cells observed in preclinical studies, intra-tumor NAD+ balance should be carefully considered in the treatment of cancer patients. Therefore, enzymes required for NAD+ synthesis and catabolism might represent potential therapeutic targets to modulate T cell functions in cancer settings.

### 3.3. Effect of PARP Inhibition on Two-Faced Janus Nuclear Factor of Activated T-Cells (NFAT)

The Nuclear Factor of Activated T-cells (NFAT) family of transcription factors plays a well-established role in T cell activation [225]. NFAT family comprises five members, of which NFAT-1, NFAT-2, and NFAT-4 are widely expressed in immune cells, playing a critical function in T cell maturation and functionality. All NFAT molecules are similarly engaged into the binding with DNA, yet showing different functions [225,226]. NFAT-1 and NFAT-2 are largely represented in activated T cells [227], where they generate a complex with AP-1 family members, c-JUN and c-FOS [228,229]. This complex migrates into the nucleus following T cell activation and calcineurin-mediated dephosphorylation [230,231], becoming the main regulator of IL-2 gene transcription.

A seminal study by Valdor et al. reported that PARP-1 is activated after T cell stimulation, but not as a consequence of DNA damage, and PARylates or directly interacts with both NFAT-1 and NFAT-2 [232]. Remarkably, PARP-1 dependent PARylation has been shown to inhibit the transcriptional activity of NFAT-1 in the human T cell line Jurkat, promoting the nuclear exit of the transcription factor, possibly due to increased phosphorylation.

Conversely, Olabisi et al. demonstrated that in mouse T cells, a direct binding and PARylation of NFAT-4 by PARP-1 positively regulates its transcriptional activity by improving the affinity for specific DNA sequences and favors T cell activation [233], although a physical interaction was also observed between PARP-1 and NFAT-1, NFAT-2, or NFAT-3.

Remarkably, NFAT-1 has been shown to elicit two parallel yet separate programs of CD8+ T cell activation and exhaustion, respectively. Muller and Rao have shown that NFAT-1 can also promote a program of T cell exhaustion by regulating the expression of key inhibitory receptors and signaling molecules able to dampen the TCR signaling, in the absence of AP-1 [234]. Accordingly, a form of NFAT-1 incompetent for association with AP-1 has been described as unable to induce a T cell effector response, yet able to promote an exhaustion molecular program [235]. On the other hand, NFAT-1-deficient CD8+ T cells failed to express either effector cytokines or the inhibitory cell surface receptors PD-1, LAG-3, and TIM-3 that are distinctive of an exhausted CD8+ T cell phenotype.

Considering the two different patterns promoted by NFAT-1 in CD8+ T cells, and the opposite effects of PARP-1 on NFAT-1 and NFAT-4 transcriptional activation described by the studies of Valdor and Olabisi, further investigations are required to dissect the effects of PARPi on the different forms of NFAT transcription factors. Moreover, since PARP-1 mediated-PARylation has been shown to inhibit the transcriptional activity of NFAT-1, it should be evaluated whether this modification could affect NFAT-1 binding with AP-1, required to induce a T cell effector response, thus favoring the T cell engagement into an exhaustion pattern. Accordingly, should this modulation be observed, it could be speculated that PARPi could provide a pharmacological selective manipulation able to contrast immune exhaustion.

### 3.4. PARPi Take Advantage of LKB1 Deficiency

Liver kinase B1 (LKB1) is a tumor suppressor involved in the control of cell response to DNA damage, encoded by STK11, a gene that is mutated in Peutz–Jeghers syndrome and in uncommon cancers [236]. Moreover, LKB1 plays a critical role in ATP preservation and interacts with BRCA1 possibly contributing to HR-mediated DNA repair [237]. Lung adenocarcinomas showing STK11/LKB1 defects, often in association with an altered KRAS [238], show poor response to immunotherapy. Indeed, lack of LKB1 has also been shown to induce accumulation of immunosuppressive adenosine in the TME, and a marked induction of HIF1A, endorsing the establishment of a “hypoxic” microenvironment in lung adenocarcinoma, characterized by a distinctive exclusion of T cells from TME and poor PD-L1 expression, defined as PD-L1-/TIL- type II cancer. The underlying mechanism involves PARP-1-mediated PARylation of STAT1, which is crucial in hampering the transcription of PD-L1 stimulated by IFNγ in LKB1 defective cells. This effect can be reversed by PARP-1 inhibition, providing the basis for combining PARPi and anti-PD-1 mAbs in LKB1 mutated lung cancer [239]. Moreover, since LKB1-deficient tumors have been shown to be enriched in terms of CD39 and CD73-related adenosine, these observations may also provide the rationale for combining PARP inhibition and CD73 blockade in this clinical setting.

### 3.5. PARP Inhibition Potentially Influences the Permanence and Function of Intra-Tumor Resident T Cells

Transforming growth factor-β (TGF-β) receptors (TβRs) deliver crucial TGF-β signaling within T cells; nevertheless, their regulation is still inadequately understood. PARP-1 has been shown to negatively regulate TβRI and II expression and, accordingly, inhibition of PARP-1 has been shown to increase both TβRI and TβRII, enhancing the sensitivity of T cells to TGFβ, although via distinct molecular mechanisms [240]. In particular, while PARP-1 selectively binds to the TβRII promoter, hampering gene transcription, its enzymatic activity is responsible for the inhibition of TβRI expression.

Because TGF-β promotes pleiotropic immune consequences [241], depending on the biological setting and cell type involved, the effects mediated by PARP inhibition on its signaling in tumors can potentially influence different aspects involving either T-cell mediated response and TME infiltration.

TGF-β is crucial for the development of FOXP3+ Tregs [242]. Therefore, PARP-1-mediated regulation of TβRs can also contribute to induce Treg generation [240], alongside with the mechanism of reduced PARylation of FOXP3 described in Section 2.1. The increase in TβRs induced by PARPi, while potentially detrimental to the generation of an effective anti-tumor response due to increased Treg generation, can also prove beneficial. In fact, TβRs may favor the expression of the residency marker CD103 on tumor-specific T cells upon engagement of TCR under tumor derived TGF-β exposure [243]. A large subset of tumor-infiltrating CD8+ T lymphocytes is represented by tissue resident (Tres) memory CD8+ T cells, that accumulate in different human cancers and are characterized by the expression of CD103 integrin and C-type lectin CD69, which both contribute to their residency features [244]. Of note, co-expression of CD103, PD-1 and CD39 has been recently shown to identify a Tres subset characterized by a distinct transcriptional signature of tumor-specific antigen responder T cells in solid tumors [245]. On the other hand, CD103+CD8+ Tres cells have been associated with better OS in various malignancies, positively affecting therapeutic sensitivity to different approaches including immunotherapy. Therefore, the sustained TβRI expression potentially mediated by PARP inhibition on intra-tumor T cells can contribute to increase the differentiation rate toward a CD103+ phenotype, distinctive of Tres, thus indirectly promoting higher intra-tumor T cell infiltration and better anti-tumor effector function.

Remarkably, PARP-1 has been shown to play a main role in the Integrin-Linked Kinase (ILK)-mediated regulation of the expression of E-cadherin [246], a large family of transmembrane or membrane-associated glycoproteins. Suppression of E-cadherin transcriptional repressors and up-regulation of E-cadherin expression have been observed as a consequence of siRNA-mediated knockdown of PARP-1, playing a critical role on the initiation of epithelial mesenchymal transition (EMT) [247]. These observations possess valuable intrinsic value as E-cadherin prevents EMT and is also crucial for binding and retaining of CD103+ Tres at the tumor site [248]. This crucial CD103-E-cadherin interaction is required for polarized exocytosis of lytic granules, mostly in the absence of ICAM-1 expression on cancer cells, leading to target cell death. Remarkably, the intra-epithelial localization of CD103+CD8+ T cells has been associated with expression of E-cadherin on tumor cells [249,250]. Indeed, recruitment of CD8+ T lymphocytes within epithelial tumor islets has been shown to be inhibited by anti-CD103 neutralizing mAbs, while TGF-β enhanced CD103-dependent T-cell movement toward epithelial tumor regions [243,251].

E-cadherin has been also identified as ligand of KLRG1 and their interaction causes the generation of a bidirectional signal in which both molecules activate downstream signaling cascades simultaneously [252], leading to integrated effects within both KLRG1 and E-cadherin–expressing cells, including DCs. KLRG1 has been considered a marker of terminal differentiation, playing an inhibitory role in human NK and T cells including effector CD8+ T cells with a highly cytotoxic and proliferative phenotype [253,254,255]. Accordingly, together with IL-7Rα, KLRG1 also identifies effector CD8+ T cell subsets with specific effector functions, migratory properties, survival, and multi-lineage memory potential [256,257]. Moreover, KLRG1 inhibition promotes the proliferation rate of A549 and H1299 lung cancer cells. Of note, KLRG1 expression has been found to positively correlate with the efficacy of ICIs, demonstrating that, besides its negative role, KLRG1 may behave as a predictive biomarker of immunotherapy clinical benefit in lung adenocarcinoma [258].

Therefore, it can be hypothesized that PARPi-mediated preservation of E-cadherin expression can enhance the development, permanence and functionality of intra-tumor Tres and the long-term survival of cytotoxic T cells, potentially improving the outcome of combined treatments with ICIs.

### 3.6. PARP Inhibition Activates AKT Kinase: Not Only a Detriment

Serine/threonine kinase AKT coordinates signals that mediate cell proliferation and survival in response to external stimuli [259]. PARPi promote AKT-mediated cytoprotective effects through a mitochondria-targeted phospho-ATM-NEMO-AKT-mTOR pathway [260]. In particular, PARP inhibition can impair PARylation of ATM, inducing its activation. In turn, ATM engages NEMO in a complex that migrates from the nucleus to the cytoplasm, where associates with mTOR and AKT, stimulating AKT-mediated survival pathways. Accordingly, a substantial and long-lasting increase in Ser473 phosphorylation of AKT has been detected by Ethier et al., following inhibition of PARP-1 by AG14361 [261]. 

Another intriguing scenario is represented by the relationship between AKT and available NAD+. Indeed, AKT can be inactivated by acetylation, a phenomenon that can be promoted by reduced expression of SIRT1, a NAD+-dependent deacetylase [262,263]. Therefore, the higher availability of NAD+ deriving from PARP-1 inhibition can also indirectly contribute to AKT activation by increasing effective SIRT1 and potentially represent another hypothetical mechanism contributing to CD8+ T cell functionality [264].

While phospho-ATM-NEMO-AKT-mTOR signalosome provides growth advantage and treatment resistance to cancer cells [265], from an immunological perspective, re-activation of AKT can support anti-tumor functional activity of T cells. Remarkably, AKT pathway controls many aspects of the anti-tumor CD8+ T cell response including the fine-tuned balance between effector and memory fate decision [266,267]. Moreover, the ability to activate the kinase is strongly reduced as T cells progress toward terminal differentiation and aging, a phenomenon accompanied by the loss of CD28 co-stimulatory molecule [146,268,269]. Phosphatidylinositol 3-kinase (PI3K)/AKT signaling pathway is activated either by TCR, CD28 and inducible costimulator (ICOS) or cytokine receptor activation [267], playing a critical role into T cell survival and activation [270,271]. Notably, inhibition of the CD28-associated PI3K/AKT pathway is the main mechanism underlying PD-1-mediated impairment of T cell activation [272]. Accordingly, as a potential consequence of DNA damage events, a combination of peptide vaccination and the DNA-methylating agent dacarbazine (DTIC) has been proved able to promote the generation of highly polyfunctional PD-1^high^ CD28^neg^Melan-A-specific CD8+ T cells, depending on AKT signaling and on the engagement of ICOS in melanoma patients [273]. Remarkably, AKT can also be stimulated in response to the other clinically used methylating compound, temozolomide [274], in an ATM and Rad3-dependent fashion [275]. Of clinical relevance, active AKT has been found only in CD28^neg^ antigen-specific CD8+ T cell clones obtained from melanoma patients who benefit from chemo-immunotherapy combined treatment, suggesting that its activation might be dependent on DNA-directed effects of alkylating agents. Therefore, similarly to what was observed for DTIC treatment, PARPi might potentially re-invigorate T cells function via AKT re-activation, either directly, or as the consequence of unresolved DNA damage [273].

### 3.7. PARP Inhibition Mimics a GSK-3 Defect

GSK-3 is a central regulator of PD-1 transcription [276], and small molecule GSK-3 kinase inhibition is as effective as PD-1 blockade in the control of tumor growth [277]. GSK-3 inhibition enhances the production of TBX21, a transcription factor which represses PD-1 transcription. This finding can contribute to explain the reduced PD-1 expression observed following PARPi [121,122,123,278]. Moreover, CD28 agonists can synergize with GSK-3α/β inhibitors to impair tumors that are resistant to anti-PD-1 immunotherapy, by significantly reducing exhaustion-related transcription factor TOX, along with co-inhibitory receptors TIM-3 and PD-1, all features suggestive of an exhausted T cell state [279]. These observations suggest a direct interference of GSK3 inhibition with the signaling pathway that is the main target of PD-1 mediated repressive activity. Therefore, besides the very promising outcome of PARPi plus GSK3 inhibitors, the impairment of GSK-3 induced by the inhibition of PARP-1 can itself directly contribute to improve the function of exhausted T cells.

Remarkably, as described previously, inhibition of GSK-3β kinase by PARPi has been shown to increase PD-L1 expression on tumor cells [118,119], irrespective of the *BRCA* gene mutational status. This effect, although obviously unfavorable per se, can increase the response to the combined PARP-1 and PD-L1 inhibition [119], fostering T cell-mediated anti-tumor response.

**Figure 2:**
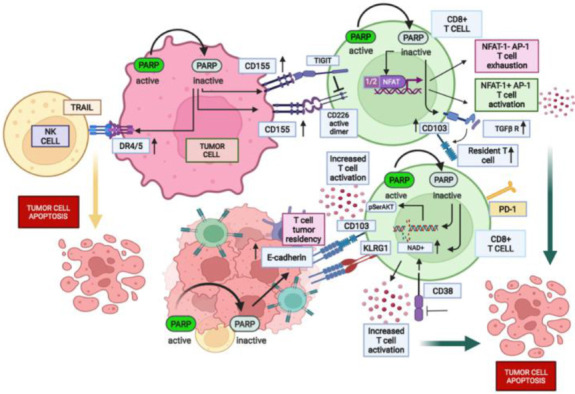
Identification of potential new targets of PARPi. Potential interference of PARPi with different features involved in anti-tumor immune response. PARPi can potentially increase: (i) expression of CD155, the ligand of both CD226 and TIGIT, interfering with the competition between co-stimulation and co-inhibition provided by these molecules; (ii) DR4/5, thus increasing the susceptibility to NK-mediated apoptosis through the engagement of TRAIL. PARPi can differently regulate the various forms of NFAT, affecting activation and/or exhaustion of T cells (see text), and can potentially induce the expression of TβR, stimulating the generation and intra-tumor permanence of Tres. Inhibition of PARP, by producing DNA damage, can favor the CD8+ T cell function by activating AKT. See text for further details. “Created with BioRender.com.”

Potential interference of PARPi with partly unexplored features involved in anti-tumor T cell response is illustrated in Figure 2.

## 4. Clinical Trials Combining PARPi with ICIs

The primary goal of cancer immunotherapy is an efficient control of cancer progression, with tumor specific cytotoxic CD8+ T cells playing a key role in the establishment of an efficient and persistent adaptive immune response. However, the efficacy of immunotherapy is still limited to a fraction of patients and the improvement of clinical outcomes is a primary goal in cancer management, especially in patients with poor intra-tumor T cell infiltration [280]. In this context, PARPi can amplify the benefit deriving from ICIs, throughout their above-described effects including preferential generation of a Th1 immune response via the cGAS-STING/type I IFN signaling, better activation of APC cells, improved intra-tumor enrolment of CD8+ cytotoxic T cells and increased PD-L1 expression on tumor cells [87,119,280].

Preclinical studies have revealed a significant synergism between PARPi and ICIs in a range of cancer settings, irrespective of BRCA1/2 deficiency [100,119,280], strengthening the hypothesis that combined treatment can represent a valuable anti-cancer approach irrespective of the DNA repair condition, with important clinical consequences [281].

Several clinical trials have assessed or are presently exploring the potential synergistic outcomes deriving from combining PARPi and ICIs in breast cancer, ovarian cancer, and others solid tumors, exhibiting interesting response rates along with manageable toxicity.

### 4.1. Completed Clinical Studies 

Lee et al. conducted the first reported phase 1 dose-escalation combination study with the anti–PD-L1 mAb durvalumab plus olaparib or the anti-angiogenic agent cediranib in 26 women with recurrent solid tumors [282]. The primary endpoint consisted of the determination of the recommended phase 2 dose, while secondary end points were response rate (RR) and pharmacokinetic parameters. Combined treatment proved tolerable and effective [282], with no dose-limiting toxicity displayed by the combination of durvalumab and olaparib and a significant 83% disease control rate (DCR). 

Efficacy and safety of olaparib and durvalumab combination in patients with solid tumors were then assessed in the MEDIOLA phase 1/2 basket clinical trial (NCT02734004), in 32 patients with different types of cancer characterized by the same *BRCA1/2* mutation [283]. Olaparib was administered for 4 weeks, followed by its combination with durvalumab until tumor progression. MEDIOLA’s main endpoints were DCR, that reached 5.6%, evaluated after 12 weeks, and tolerability. Seven complete responses were observed, and the overall response rate (ORR) was 71.9%. Grade 3 or 4 adverse effects were represented by anemia, neutropenia, and pancreatitis [284], although the combination was in general well tolerated, providing a potential therapeutic option for the management of ovarian and breast cancer, along with other tumors with *BRCA1/2* mutations.

Promising outcomes were revealed in the TOPACIO open-label, single-arm phases 1 and 2 trial (NCT02657889), including 62 patients, and comparing the effects of a combination including pembrolizumab and niraparib, regardless of the *BRCA* mutation status [285,286]. The primary endpoints of phase 1 study were the assessment of dose-limiting toxic effects as well as recommended phase 2 dose and dosage plan, while the primary goal of phase 2 was to evaluate either partial or complete response rates. The integrated analysis of phase 1 results in ovarian cancer and TNBC patients and of phase 2 outcomes in ovarian cancer patients showed that ORR and DCR were 18% and 65%, respectively. Interestingly, patients with non-mutated *BRCA* tumors showed better outcomes with the combination as compared with either agent used as monotherapy.

The efficacy and safety of combined niraparib and pembrolizumab was estimated in an open-label, single-arm phase 2 study (NCT02657889) enrolling patients with advanced or metastatic TNBC, in the presence or absence of *BRCA* mutations and regardless of PD-L1 expression [287]. The primary endpoint was ORR, while among secondary endpoints were safety, DCR, duration of response (DOR), PFS, and OS. Remarkably, among the 47 individuals evaluable for treatment efficacy, ORR and DCR, analyzed in 21% and 49% of patients respectively, were 90%, with better results obtained in those with *BRCA*-mutated tumors.

The investigational PARPi pamiparib in combination with the anti PD-1 mAb tislelizumab was tested in a phase 1a/b clinical trial (NCT02660034), performed on 49 patients bearing different types of formerly treated advanced solid tumors [288]. The primary endpoint of the phase 1a dose-escalation study was tolerability, indicative of the optimal dose to be tested in phase 2. At a mean follow-up of 8.3 months, patients showed 20% objective responses (OR). The combination showed good tolerability. In particular, no grade 5 adverse effects were observed, while hepatitis and autoimmune hepatitis were the only serious adverse events, occurring in 8% of patients. The most frequent grade 3 adverse effect was anemia, observed in 12% of patients, thus providing the rationale for additional explorations of the combined approach [288].

An interesting single-center, proof-of-concept phase 2 study involving olaparib plus durvalumab, conducted on 35 patients with ovarian cancer mostly platinum-resistant and *BRCA* wild-type, among other solid tumors (NCT02484404), has provided a link between the clinical outcome and biochemical modifications occurring within the TME. Matched fresh core biopsies and blood samples have been collected to test the hypothesis that the PARPi promotes an immunostimulatory microenvironment in ovarian cancer thus complementing IC blockade [289]. DCR following treatment was 71%, in association with improved IFNγ production, CXCL9/CXCL10 chemokine expression and increased intra-tumor T cell infiltration, all representative of an immune-stimulatory environment. Remarkably, increased IFNγ production was associated with improved PSF, while elevated VEGFR-3 levels were associated with worse outcome, prompting the authors to suggest that further co-blockade of VEGF/VEGFR pathway could improve the efficacy of the combination treatment.

The phase 3 randomized JAVELIN Ovarian PARP 100 trial (NCT03642132) [290], performed on 720 patients, analyzed the combined use of talazoparib and the anti-PD-L1 mAb avelumab as maintenance therapy following avelumab plus chemotherapy, in previously untreated epithelial ovarian cancer patients. Unfortunately, the trial was discontinued based on the results of interim analysis indicating that the study would not have met its primary endpoint of PFS (Merck KGaA, Darmstadt, Germany, and Pfizer, Press Release, 19 March 2019).

Notwithstanding the elevated tumor mutational load, IC blockade shows inadequate efficacy in SCLC. Thus, Thomas et al., by postulating that PARPi might increase SCLC vulnerability to IC blockade [291], conducted a single-arm, phase 2 trial (NCT02484404), recruiting 20 patients with relapsed, platinum-resistant/refractory disease who received a combination of durvalumab and olaparib, with PFS as primary endpoint. Remarkably, a parallel research integrated biopsy collection, to evaluate whether the combination could improve the SCLC immunoscore. Clinical benefit was observed in 21.1% of patients, although tumor inhibition was witnessed only in patients with an inflamed phenotype. Therefore, the combination did not meet the pre-specified endpoint for efficacy yet implying that tumor immune phenotypes may be relevant for SCLC responses to IC blockade combinations. However, as suggested by the authors, the predictive value of pre-existing CD8+ T-cell infiltrates observed in this study needs to be confirmed in larger cohorts.

Altogether, the described data suggest that PARPi in combination with ICIs are well-tolerated, stimulating further clinical investigation to establish their clinical efficacy.

### 4.2. Ongoing Phase 3 Clinical Studies

Several ongoing phase 1–3 trials are currently exploring the combination of PARPi with IC blockade by using approved anti-PD-1/PD-L1 mAbs or novels agents such as TSR-022 (cobolimab) that targets the co-inhibitory molecule TIM-3.

Avelumab in combination with talazoparib is currently examined in patients with locally advanced (primary or recurrent) or metastatic solid tumors with a *BRCA1/2* or *ATM* gene defect, respectively, enrolled in two parallel cohorts within a single-arm phase 2 study (NCT03565991) [292]. Primary endpoint of the study is OR, while among secondary endpoints are time to tumor response (TTR), DR, PFS, OS, time to prostate-specific antigen (PSA) progression for castration-resistant metastatic prostate cancer (mCRPC) patients, and pharmacokinetic parameters.

Phase 3 trials are critical for determining the clinical potential of novel combined therapies, identifying the best therapeutic combinations along with biomarkers to be integrated into the clinical study design as predictors of clinical response. Ongoing phase 3 studies combining PARPi with ICIs are summarized in Table 1.

A number of phase 3 investigations are currently evaluating the use of PARPi as maintenance therapy in association with ICIs for the management of ovarian cancer. Based on the promising efficacy obtained with niraparib and pembrolizumab in patients with platinum-resistant or secondary refractory ovarian cancer irrespective of biomarker grade [286], the phase 3 ENGOT-OV44/FIRST trial (NCT03602859) is comparing the PFS of patients with FIGO stage 3/4, non-mucinous, epithelial ovarian cancer treated with standard therapy (paclitaxel and carboplatin ± the anti-VEGF-A mAb bevacizumab, SOC) or with SOC plus the anti PD-1 mAb dostarlimab and niraparib. Remarkably, after 1 cycle of SOC, patients are stratified by bevacizumab use, *BRCA* mutational/HR status and disease burden before randomization. A distinctive feature of this investigation is the possibility to introduce additional therapeutic adjustments while new information about the patient’s biomarker response is provided [293].

First-line olaparib maintenance therapy after pembrolizumab plus chemotherapy treatment is being investigated in ENGOT-OV43/KEYLYNK-001 (NCT03740165), a phase 3, randomized, double-blind study performed in stage III/IV *BRCA*-wild-type epithelial ovarian cancer, primary peritoneal cancer, or fallopian tube cancer patients, to evaluate PFS and OS [294]. In parallel, the phase 3 international randomized, ATHENA trial (NCT03522246), is currently evaluating whether a combination of rucaparib with nivolumab as maintenance treatment may extend PFS compared to rucaparib alone following standard platinum-based chemotherapy in newly diagnosed stage III/IV ovarian cancer patients [295]. Patients have been selected irrespective of tumor *BRCA* mutational status or HR defect. The study, while providing useful clinical information on the combined approach as maintenance treatment, also aims at providing feasible biomarkers of prediction of both response and resistance to the treatment.

Based on the observations obtained by Lampert et al. in a proof-of-concept phase 2 study [289], the double-blind, randomized, phase 3 DUO-O investigation (NCT03737643) [296], still recruiting at the time of writing of the present manuscript, is evaluating the efficacy and safety of combined durvalumab and olaparib maintenance treatment in newly diagnosed advanced ovarian cancer patients. The primary endpoint of the study is PFS, and secondary endpoints are OS, OR and DOR. Patients are being randomized according to the tumor *BRCA* mutation status and treated with platinum-based chemotherapy and bevacizumab with or without durvalumab as conditioning therapy followed by durvalumab and bevacizumab, plus or minus olaparib as maintenance treatment.

The randomized multicenter phase 3 NItCHE trial (MITO 33) (NCT04679064) is evaluating the efficacy of a combination including niraparib and dostarlimab in increasing OS, PFS along with the time interval required for additional therapy, compared to chemotherapy alone, in recurrent ovarian, fallopian tube, or primary peritoneal cancer patients who had received no more than two previous treatments with PARPi and/or ICIs [297].

To establish whether maintenance therapy including niraparib following anti PD-L1 mAb atezolizumab plus carboplatin can ameliorate PFS as compared with placebo, a randomized, phase 3 double-blinded trial, the Atezolizumab and Niraparib Treatment Association (ANITA, NCT03598270), is being conducted in recurrent ovarian, tubal, or peritoneal cancer patients, treatment-free for at least 6 months, with PFS as primary endpoint [298].

A number of studies are also currently evaluating PARPi in the maintenance therapy of endometrial cancer. In particular, ROCSAN is a multicenter, randomized, open-label, integrated phase 2/3 study comparing the efficacy of niraparib or niraparib plus dostarlimab vs. chemotherapy for the treatment of endometrial/ovarian carcinosarcoma after at least one line of platinum-based chemotherapy [299]. After the phase 2, patients with recurrent or progressing endometrial or ovarian cancer, following at least a first line of platinum-based chemotherapy, are being randomized to receive either niraparib as monotherapy or niraparib in combination with dostarlimab or standard chemotherapy (paclitaxel, doxorubicine, gemcitabine, and topotecan). The primary objective of the phase 2 is to choose the best treatment between niraparib and dostarlimab/niraparib based on RR at 4 months, to be used in phase 3 vs standard chemotherapy. Secondary endpoints include safety, PFS, PFS2, TTST, ORR, DOR, and patient-reported outcome. The trial is also testing tumor response/resistance in relationship to the immune environment features and genetic instability of the tumor.

The effect of the combination of olaparib and durvalumab as maintenance therapy following durvalumab to carboplatin and paclitaxel on the PFS in patients with stage III/IV or recurrent endometrial cancer is being examined in the multicenter, double-blind, phase 3 DUO-E trial (NCT04269200) [300]. Primary and secondary endpoints are PFS and OS, respectively, of the combined maintenance treatment as compared to durvalumab alone.

The second part of the RUBY trial (ClinicalTrials.gov Identifier: NCT03981796) has been also designed to evaluate the efficacy and safety of PARPi as maintenance therapy in endometrial cancer setting. In particular, the use of dostarlimab in combination with carboplatin-paclitaxel followed by dostarlimab plus niraparib vs. dostarlimab plus placebo is being investigated in recurrent or primary advanced endometrial cancer, according to MSI-H or microsatellite stable. The primary endpoint is PFS assessed by investigators, while secondary efficacy endpoints are PFS assessed by blinded independent central review, OS, ORR, DOR, DCR, safety and tolerability, and patient-reported outcomes.

There is a crucial requirement for a rational exploration of possible therapeutic schedules and combinations for the management of TNBC. Remarkably, the use of PARPi in the treatment of TNBC has been evaluated in numerous clinical investigations, as extensively described by Singh et al. [301]. To identify an effective maintenance therapeutic option for TNBC, able to circumvent possible detrimental effects or resistance as consequence of extended chemotherapy, the randomized phase 2/3 investigation KEYLYNK-009 (NCT04191135) is comparing the combined use of pembrolizumab and olaparib to the combination of pembrolizumab plus chemotherapy, as maintenance treatment following induction therapy with pembrolizumab and chemotherapy in locally recurrent, inoperable TNBC [302], by analyzing PFS and OS, along with quality of life and safety.

NSCLC cells characterized by defects in HR genes have been shown to respond to PARPi [303], providing the rationale for testing them in combination with ICIs, especially in the absence of any actionable mutations (according to the NCCN Clinical Practice Guidelines in Oncology: Non-Small Cell Lung Cancer.Version 5.2021).

The phase 3 randomized, double-blind ZEAL-1L trial (NCT04475939) is currently comparing the efficacy and safety of maintenance therapy with niraparib and pembrolizumab against placebo plus pembrolizumab in Stage IIIB–IV NSCLC patients in the absence of recognized mutations following response to platinum and pembrolizumab [304]. Primary endpoints are represented by PFS and OS, while a main secondary endpoint is the time to progression in the central nervous system, along with PFS and OS by the PD-L1 status, quality of life, safety, pharmacokinetics, etc. 

The KEYLYNK-006 (NCT03976323) is a randomized, phase 3, open-label trial currently analyzing the outcome of first line pembrolizumab plus pemetrexed/platinum followed by pembrolizumab plus olaparib compared to pembrolizumab plus pemetrexed as maintenance therapy in metastatic, nonsquamous NSCLC patients [305]. Primary endpoints are PFS and OS, while secondary endpoints are represented by safety and quality of life. Experimental endpoints are ORR and response interval.

The use of pembrolizumab with or without olaparib vs chemoradiation therapy (CCRT) followed by durvalumab as maintenance therapy after pembrolizumab plus CCRT is being evaluated in the randomized phase 3 KEYLYNK-012 trial (NCT04380636) in patients with unresectable, locally advanced, stage III NSCLC [306]. PFS and OS are dual primary endpoints, while secondary endpoints include ORR, DOR, safety, and quality-of-life.

The efficacy of olaparib and pembrolizumab as maintenance therapy vs. olaparib or placebo following pembrolizumab plus carboplatin and paclitaxel is presently being explored in the multicenter KEYLYNK-008 clinical trial (NCT03976362) also in the setting of squamous metastatic NSCLC (sqNSCLC) [307]. PFS and OS are the trial endpoints, safety and patient-testified outcomes are secondary endpoints, while ORR and response interval represent exploratory endpoints.

The KEYLYNK-010 (NCT03834519) is a phase 3 trial currently assessing the efficacy and safety of olaparib plus pembrolizumab in mCRPC patients pre-treated with enzalutamide or abiraterone that had progressed following docetaxel treatment [308]. The primary endpoints are OS and PFS, while secondary endpoints are the time interval to PSA progression, first skeletal symptomatic event, radiographic evidence of progression at the soft tissue and pain, and requirement for subsequent treatment. Of note, this study has introduced the identification of molecular biomarkers indicative of therapeutic response as exploratory endpoints.

Finally, the long-term safety association of the anti-PD-1 mAb tilslezumab with pamiparib, a selective PARP-1/2 recently approved in China [309] for the management of germline *BRCA* mutated recurrent advanced ovarian, fallopian tube, or primary peritoneal cancer resistant to previous lines of chemotherapy is currently being evaluated for the treatment of different advanced malignancies [NCT04164199], including ovarian, fallopian tube, peritoneum, pancreas, and breast cancer, in patients who had participated to a previous phase 1a/b study [288,310]. Primary endpoint of the study is the identification of adverse events, while secondary outcome is OS. 

Scarce information is available about combined PARP inhibition and CTLA-4 blockade, although showing good tolerability in pretreated ovarian cancer patients [281]. In particular, phase 1 investigation combining olaparib and tremelimumab has delivered positive therapeutic indication, supporting the phase 2 NCT02571725 study [311].

## 5. Considerations about the Use of PARPi for Hematological Malignancies

The advantages and drawbacks about a possible use of PARPi for the treatment of hematological malignancies have been recently extensively reported by Skelding and Lincz [312]. The use of PARPi was not originally assessed for the treatment of hematological malignancies due to the very low frequency of *BRCA1/2* mutations in these tumors [313]. Moreover, the occurrence of secondary myeloid malignancies has been described in solid cancer patients treated with PARPi, fostering doubts about their possible use for the treatment of hematological malignancies. However, since besides BRACA1/2 defects, additional deficiencies involving the DNA repair machinery might also provide a ‘BRCAness’ phenotype and be predictive of PARPi sensitivity, PARPi might play a role in the management of distinctive blood cancers, defective in HR and DSBs repair [312,314].

Several preclinical investigations, although providing conflicting observations, have shown how olaparib, veliparib, rucaparib, and talazoparib may induce synthetic lethality in acute leukemia cells when combined with different chemotherapeutic agents or irradiation, as described by Skelding and Lincz [312]. Accordingly, veliparib, alone or in combination with chemotherapy, has provided encouraging clinical results in the context of early clinical trials performed mainly on AML, myeloproliferative neoplasms (MPN) and lymphoma patients, showing good tolerability and, when assessed, 16–25% response in AML patients [312]. Of note, experimental observations have suggested that high levels of H2AX histone phosphorylation induced by the treatment might represent the best predictor of favorable clinical outcome [315,316]. Additional studies on the genetic background associated to defective DNA repair are strongly required to better identify those leukemia patients who can benefit from PARPi-based treatments. Moreover, further investigation is also needed to assess whether PARPi can contribute to the establishment of an immunological “hot” microenvironment in hematological malignancies, providing the required rationale for a combined treatment with immunostimulatory approaches. In particular, AML cells have been shown to induce apoptosis of NK and T cells through a mechanism dependent on PARP-1 [317]. Moreover, as described in Section 2.2 of the present Review, PARP-1 suppresses the expression of NKG2D ligands in leukemia stem cells, therefore contributing to their escape from immune surveillance by NK cells [158]. Accordingly, by increasing the expression NKG2D ligands on tumor cells [158], PARPi can potentially contribute to foster the function of NK cells and activated CD8+ T cells, further highlighting the feasibility of combining PARPi and immunostimulatory therapies for the treatment of AML.

## 6. Delivery of PARPi at the Tumor Site: Nanoparticle-Based Formulation

Remarkably, PARPi delivery nanosystems, through a fine-measured delivery, could meet the requirement for an improved drug accumulation at the tumor site, reducing undesired adverse effects, while positively influencing tumor specific responses. The principal nanosystem approaches for conveying PARPi, either as monotherapy or in association with chemotherapy or radiotherapy, have been extensively described by Cai et al. [318]. This study indicates that PARPi treatment based on nanotechnology strategies can provide synergistic benefits when used in combined treatments in cancer management. Of note, a nano-liposome formulation of talazoparib has been recently shown to induce complete regression in mammary tumors of BRCA-deficient mice, while strongly improving tumor-associated immune cell populations [319]. These observations provide the required immunological and clinical foundations for a promising treatment combining a nano-liposome PARPi formulation and PD-1/PD-L1 blockade [319].

## 7. Conclusions

Preclinical observations strongly support the assumption that PARPis might prime cancers to respond to IC blockade. These findings have been confirmed by the results deriving from early clinical studies, suggesting that the combination of PARPi with anti-PD-1/PD-L1 mAbs is well tolerated and deserves further investigation to evaluate its clinical efficacy, especially in the setting of HR-defective tumors.

Most of the phase 3 clinical trials testing the PARPi combination with ICIs are still ongoing and several crucial issues are still open questions, including the actual clinical advantage over single-agent therapies, along with the best drug combination and treatment schedule to be used for the different tumor types. To this end, an implementation of phase 2 randomized trials is strongly required to circumvent the limitations of insufficiently informative single-arm phase 2 investigations, based on historical controls [320]. This approach can potentially provide more informative results to design the following phase 3 studies including those testing novel combined therapies.

Based on the proposed mechanisms described in the previous sections, PARPi endowed with the highest PARP trapping efficiency and consequently with the highest ability to damage DNA likely represent the best candidates for being combined with ICIs. Results from the currently ongoing clinical studies will better elucidate the therapeutic potential of this combination, although the identification of immunological biomarkers indicating patients who can benefit from this therapeutic approach is still an unmet medical need. However, since most of the ongoing clinical trials analyze the use of PARPi in combination with ICIs as maintenance therapy, the assessment of the real benefit deriving from the treatment cannot be promptly determined, as patients might be disease-free or have minimal residual disease. This will require the analysis of long-term primary and secondary endpoints along with the suitable exploratory endpoints that can provide a more specific assessment of the individual patient’s response, according to the expression of specific molecular biomarkers. As discussed by Stewart et al. [321], in patients with inadequate sensitivity to PARPi, such as those with proficient DNA DSBs repair, RR may represent a useful endpoint. RR can be also an appropriate endpoint for cancer types normally treated with anti-PD-1/-PD-L1 mAbs, such as NSCLC, especially in the case of PD-L1-negative or -low-expressing tumors. Indeed, additional effective treatments for NSCLC patients progressing after anti-PD-1/-PD-L1 mAbs-based therapies are urgently needed. This issue is being addressed by the platform HUDSON trial (NCT03334617), a biomarker-directed, phase 2 study. Conversely, a long-term assessment of improved DOR and OS seems more suitable to determine the therapeutic advantage of the drug combination in patients responsive to PARPi, such as in the case of *BRCA*1/2-mutant or otherwise HR-defective ovarian cancer.

What emerges from the preclinical investigation is that PARPi can potentially influence numerous features and signaling pathways of distinctive immune cell subsets regulating the anti-tumor T cell activity. Therefore, further studies are strongly required to dissect the involvement of PARP in modulating the immune response in distinctive tumor settings and to identify unexplored and possibly patient’s specific immune features that might represent pharmacological targets of PARPi. However, since DNA damage seems to be a main trigger for PARPi-mediated modulation of immune responses, it is expected that HR-deficient tumors with innate resistance to PARPi will also fail to respond to their combination with ICIs. Overall, the unveiling of the different mechanisms of resistance to PARPi and ICIs is a prerequisite to encourage the use of new combined approaches, besides the choice of the optimal treatment dose and schedule for both agents, to increase clinical benefit and reduce adverse effects. About the latter aspect, local administration of PARPi might allow an improved drug delivery at the tumor site with decreased systemic toxicity.

In the search of additional predictive markers of response to PARPi, several studies have demonstrated that PARP-1 expression levels strongly influence the cytotoxic effects of PARPi [322,323,324]. Therefore, noninvasive techniques to measure PARP-1 expression levels are expected to improve patients’ selection for PARPi-based therapies. In this regard, encouraging results derive from a PET imaging study aimed at quantifying PARP-1 expression in 13 breast cancer patients using a radiolabeled tracer with a chemical structure similar to PARPi [325]. Tracer uptake at baseline was an indicator of PARP-1 expression, whereas abrogation of the signal after PARPi administration likely reflected drug-target interaction. Interestingly, a patient showing a minimal uptake before and after treatment had disease progression on PARPi, suggesting that PARP-1 expression might represent a potential predictive marker that deserves further clinical investigation.

Indeed, the analysis of both nature and extent of DDR defects, not limited to *BRCA1/2* germline and somatic, deleterious mutations but including a detailed characterization of the tumor mutational signature, is critical for reaching appropriate therapeutic decisions and for patients’ stratification according to the PARPi sensitivity of their tumors. Nevertheless, the observation that cancer patients with non-mutated *BRCA* and non-defective HR might derive higher benefit from PARPi-ICIs combination (ORR 19%) than expected from either agent as monotherapy (ORR 0-4% or 4-10% with PARPi or anti-PD-1/PD-L1 mAbs, respectively) [286], suggests that additional mechanisms (e.g., modulation of gene transcription) not directly related to PARPi-induced DNA damage might account for PARPi/ICIs synergism. There is still a long way to go, and further studies are required to detect molecular and immunological predictive biomarkers as well as additional PARPi-modulated functions both in tumor cells and in the immunological components of the TME. The results of these studies can provide useful insights to identify the most appropriate immunotherapeutic strategy that, in a combined approach, might result in the best outcome for each individual patient according to the current model of precision medicine.

## Figures and Tables

**Table 1:** Phase 3 clinical trials combining PARPi with ICIs.

PARPi	ICI	Trial	Combinations Including PARPi	Identifier—Reference
talazoparib	avelumab (anti PD-L1)	JAVELIN ovarian PARP 100	Maintenance therapy with talazoparib plus/minus avelumab following avelumab plus chemotherapy in untreated advanced ovarian cancer	NCT03642132 [290]
niraparib	dostarlimab (anti PD-1)	ENGOT-0V44/FIRST	First-line treatment with niraparib plus platinum-based therapy and dostarlimab vs. SOC platinum-based therapy of advanced non-mucinous epithelial ovarian cancer	NCT03602859 [293]
olaparib	pembrolizumab (anti PD-1)	ENGOT-OV43/ KEYLYNK-001	Maintenance therapy with olaparib plus/minus pembrolizumab following first line carboplatin/paclitaxel in BRCA-wild-type epithelial ovarian cancer	NCT03740165 [294]
rucaparib	nivolumab (anti PD-1)	ATHENA	Maintenance therapy with rucaparib and nivolumab following response to front-line treatment in newly diagnosed ovarian cancer	NCT03522246 [295]
olaparib	durvalumab (anti PD-L1)	DUO-O	Maintenance therapy with olaparib plus bevacizumab and durvalumab, preceded by platinum-based chemotherapy plus bevacizumab in newly diagnosed ovarian cancer	NCT03737643 [296]
niraparib	dostarlimab (anti PD-1)	NItCHE- MITO33	Niraparib plus dostarlimab vs. chemotherapy in recurrent ovarian, fallopian tube or primary peritoneal cancer	NCT04679064 [297]
niraparib	atezolizumab (anti PD-L1)	ANITA	Maintenance with niraparib plus/minus atezolizumab following platinum-based chemotherapy plus/minus atezolizumabin in recurrent ovarian, tubal or peritoneal cancer	NCT03598270 [298]
niraparib	dostarlimab (anti PD-1)	ROCSAN	Niraparib plus dostarlimab and niraparib alone vs. chemotherapy in recurrent ovarian carcinosarcoma	NCT03651206 [299]
olaparib	durvalumab (anti PD-L1)	DUO-E	Maintenance therapy with durvalumab plus/minus olaparib after first line treatment in advanced and recurrent endometrial cancer	NCT04269200 [300]
niraparib	dostarlimab (anti PD-1)	RUBY part 2	Niraparib plus dostarlimab after dostarlimab and chemotherapy in recurrent or primary advanced endometrial cancer	NCT03981796
olaparib	pembrolizumab (anti PD-1)	KEYLYNK-009	Maintenance with olaparib plus pembrolizumab vs. chemotherapy plus pembrolizumab after first-line chemotherapy plus pembrolizumab in TNBC	NCT04191135 [302]
niraparib	pembrolizumab (anti PD-1)	ZEAL-1L	Maintenance therapy with pembrolizumab plus/minus niraparib following SOC first-line platinum-based chemotherapy and pembrolizumab in advanced/metastatic NSCLC	NCT04475939 [304]
olaparib	pembrolizumab (anti PD-1)	KEYLINK-006	Pembrolizumab plus maintenance olaparib vs. pembrolizumab plus maintenance pemetrexed in non-squamous NSCLC	NCT03976323 [305]
olaparib	pembrolizumab (anti PD-1)	KEYLYNK-012	Pembrolizumab and CCRT followed by maintenance with pembrolizumab plus/minus olaparib placebo vs. CCRT followed by durvalumab in NSCLC	NCT04380636 [306]
olaparib	pembrolizumab (anti PD-1)	KEYLYNK-008	Maintenance therapy with pembrolizumab plus/minus olaparib in first line squamous NSCLC	NCT03976362 [307]
olaparib	pembrolizumab (anti PD-1)	KEYLYNK-010	Pembrolizumab plus olaparib vs. hormone therapy in mCRPC unselected for HR repair defects resistant to hormone/chemotherapy	NCT03834519 [308]
pamiparib	tislelizumab (anti PD-1)	-	Tislelizumab plus or minus pamiparib in advanced malignancies	NCT04164199
